# World-wide research architecture of vitamin D research: density-equalizing mapping studies and socio-economic analysis

**DOI:** 10.1186/s12937-018-0313-6

**Published:** 2018-01-06

**Authors:** Dörthe Brüggmann, Annahita Alafi, Jenny Jaque, Doris Klingelhöfer, Michael H. Bendels, Daniela Ohlendorf, David Quarcoo, Frank Louwen, Sue A. Ingles, Eileen M. Wanke, David A. Groneberg

**Affiliations:** 1 Department of Gynecology and Obstetrics, Goethe-University, Frankfurt, Germany; 2 Division of Preventive Medicine, Institute of Occupational Medicine, Social Medicine and Environmental Medicine, Goethe-University, Frankfurt, Germany; 3 Department of Obstetrics and Gynecology, Keck School of Medicine, University of Southern California, California, Los Angeles USA; 4 Department of Preventive Medicine, University of Southern California, Los Angeles, USA

**Keywords:** Vitamin D, Density equalizing mapping, Research architecture

## Abstract

**Background:**

Despite the numerous associations of vitamin D with health and disease, vitamin D deficiency is still common from a global perspective. While basic research, clinical and preventive activities grow constantly in vitamin D research, there is no in-depth analysis of the related global scientific productivity available so far.

**Methods:**

Density equalizing mapping procedures (DEMP) were combined with socioeconomic benchmarks using the NewQIS platform.

**Results:**

A total of 25,992 vitamin D-related research articles were identified between 1900 to 2014 with a significant increase (r^2^ = .6541) from 1900 to 2014. Authors located in Northern America – especially in the USA – distributed the majority of global vitamin D research, followed by their Western European counterparts. DEMP-analysis illustrates that Africa and South America exhibit only minor scientific productivity. Among high-income group countries, Scandinavian nations such as Denmark or Finland (2147.9 and 1607.7 vitamin D articles per GDP in 1000 billion USD) were highly active with regard to socioeconomic figures.

**Conclusion:**

Networks dedicated to vitamin D research are present around the world. Overall, the Northern American and Western European nations occupy prominent positions. However, South American, African and Asian countries apart from Japan only play a minor role in the global research production related to vitamin D. Since vitamin D deficiency is currently increasing in the Americas, Europe and parts of the Middle East, research in these regions may need to be encouraged.

## Background

Dietary aspects of micronutrients play a major role in health and disease. Vitamin D and its metabolites occupy an important role in many biological processes relevant in tissue homeostasis and metabolism. Hence, a sufficient vitamin D status is crucial for musculoskeletal and extraskeletal health [1–4]. As elegantly summarized by Zhang and Naughton vitamin D deficiency is a common condition worldwide, especially during winter months [5]. Globally, inhabitants of South Asia and the Middle East have a higher prevalence of lower vitamin D concentrations (less than 25 nmol/L) across all ages than their counterparts in the rest of the world [6].

Although overt vitamin D deficiency is rare in developed countries, subclinical forms occur frequently and are associated with the pathogenesis and progression of neoplasms, hypertension, diabetes, immune disorders such as multiple sclerosis, musculoskeletal conditions, as well as osteoporosis [5]. These epidemiological findings have public health relevance since low vitamin D concentrations are highly prevalent among populations living in high latitudes, mainly indoors, and among those who are older or dark skinned [7]. In this context, national food fortification programmes seem a promising strategy as implemented in Finland by national decrees in 2002 and 2010 [8].

Due to these considerations, vitamin D merits awareness among researchers from many biomedical specialities, and policy makers, as well as public health and healthcare professionals [5]. Numerous scientific questions remain, and more research is required to find answers in the areas of basic science, epidemiology and public health. Globally, funds need to be dedicated to vitamin D research, and future research projects have to be planned according to identified shortcomings. In view of the large number of existing scientific publications related to vitamin D we aimed to assess the global research output and associated scientific networks related to this micronutrient. For this study, we used a previously established study protocol, which combines bibliometric tools and modern visualization techniques including density equalizing mapping [9–11].

## Methods

### NewQIS platform

As in recent studies, we employed the New Quality and Quantity Indices in Science (NewQIS) platform to assess the global research productivity on vitamin D regarding quantitative and qualitative aspects, geographical and chronological developments, research networks and socio-economic indicators [12]. NewQIS is an international and multidisciplinary project that combines scientometric tools with density equalizing calculations to evaluate and visualize research activity and quality for the purpose of benchmarking processes [13, 14]. It was started  in 2008–9, and since then, this approach has been extensively used to dissect the global research output in specific fields of science and medicine ranging from public health and health policy issues [15–18], to infectious diseases [19], respiratory medicine [20], and tobacco control [13, 21]. This platform uses established and standardized techniques for data analysis, which allows the reliable comparison of results generated here to findings of previous NewQIS studies on other diseases and related biomedical fields.

### Data source

NewQIS is able to extract data from both PubMed and Web of Science (WoS, Thomson Scientific) databases. For this study, we preferred the WoS. This database offers the advantagous feature of automated ‘citation analysis’ of its indexed publications [22–24].

### Search strategy and timeframe

The following search-term was used: **TITLE:** (“*vitamin* d” OR “*vitamin* d1” OR “*vitamin* d2” OR “*vitamin* d3” OR “*vitamin* d4” OR “*vitamin* d5”) *OR* **TITLE:** (*cholecalcifero* OR ergocalcifero* OR ergosterin OR calcitriol OR calciol) *OR* **TITLE:** (1,25(OH)2D3 OR 1,25(OH)2D2 OR antiricketic OR antirachitic) *OR* **TITLE:** (dihydrotachysterol OR calcipotriol OR alfacalcidol OR Paricalcitol OR tacalcitol) *OR* **TITLE:** (“*vitamin* d” OR “*vitamin* d-1” OR “*vitamin* d-2” OR “*vitamin* d-3” OR “*vitamin* d-4” OR “*vitamin* d-5”).

This term was entered into the WoS search field to identify the total number of published items on Vitamin D and its metabolites. We conducted a “title” search to minimize the identification of off-topic publications and maximize the validity of our search. Additionally, the entries were filtered by document type, only the original articles were analysed. The time frame was set from 01/01/1900 to 12/31/2014. We did not regard research productivity in the years from 2015 onwards since data on citation rates would be incomplete at the time data acquisition was performed for this study.

The research was further refined by WoS categories. Articles listed in the following categories were included: ALLERGY OR ANATOMY MORPHOLOGY OR ANESTHESIOLOGY OR CARDIAC CARDIOVASCULAR SYSTEMS OR CHEMISTRY MEDICINAL OR CLINICAL NEUROLOGY OR CRITICAL CARE MEDICINE OR DENTISTRY ORAL SURGERY MEDICINE OR DERMATOLOGY OR EMERGENCY MEDICINE OR ENDOCRINOLOGY METABOLISM OR ENGINEERING BIOMEDICAL OR GASTROENTEROLOGY HEPATOLOGY OR GENETICS HEREDITY OR GERIATRICS GERONTOLOGY OR GERONTOLOGY OR HEALTH CARE SCIENCES SERVICES OR HEALTH POLICY SERVICES OR HEMATOLOGY OR IMMUNOLOGY OR INFECTIOUS DISEASES OR INTEGRATIVE COMPLEMENTARY MEDICINE OR MEDICAL INFORMATICS OR MEDICAL LABORATORY TECHNOLOGY OR MEDICINE GENERAL INTERNAL OR MEDICINE LEGAL OR MEDICINE RESEARCH EXPERIMENTAL OR NEUROSCIENCES OR NURSING OR NUTRITION DIETETICS OR OBSTETRICS GYNECOLOGY OR ONCOLOGY OR OPHTHALMOLOGY OR ORTHOPEDICS OR OTORHINOLARYNGOLOGY OR PARASITOLOGY OR PATHOLOGY OR PEDIATRICS OR PERIPHERAL VASCULAR DISEASE OR PHARMACOLOGY PHARMACY OR PHYSIOLOGY OR PRIMARY HEALTH CARE OR PSYCHIATRY OR PSYCHOLOGY OR PSYCHOLOGY BIOLOGICAL OR PSYCHOLOGY CLINICAL OR PSYCHOLOGY DEVELOPMENTAL OR PSYCHOLOGY EXPERIMENTAL OR PSYCHOLOGY MULTIDISCIPLINARY OR PUBLIC ENVIRONMENTAL OCCUPATIONAL HEALTH OR RADIOLOGY NUCLEAR MEDICINE MEDICAL IMAGING OR REHABILITATION OR RESPIRATORY SYSTEM OR RHEUMATOLOGY OR SOCIAL SCIENCES BIOMEDICAL OR SUBSTANCE ABUSE OR SURGERY OR TRANSPLANTATION OR TROPICAL MEDICINE OR UROLOGY NEPHROLOGY OR VIROLOGY). The articles were restricted to biomedical research by excluding the WoS categories that are related to veterinary medical, agricultural or zoological aspects. Regarding the searched databases, the Art & Humanities Citation Index was excluded from our search.

### Data analysis and categorization

As previously described for NewQIS studies on osteoporosis and other diseases [19, 24], all published items referring to vitamin D were analysed related to publication date, country of origin, source title, and author. The number of citations and the average citations per item (citation rate) were calculated [19, 24]. We illustrated the results in diagrams and density-equalizing maps. The underlying density-equalizing mapping procedures (DEMP) are based on the algorithm of Gastner and Newman [25], and used to visualize the distribution of the total number of vitamin D related publications and the average citation rate of vitamin D-related publications in a country specific manner [25]. Using this approach, separate territories (countries) were resized in proportion to selected variables (e.g. numbers of published items or the average citation rate of vitamin D-related publications).

### Analysis of cooperation

International collaborative activities were analysed as earlier described [19, 22, 24]. If at least two authors came from different countries and contributed to a publication, this relationship was defined as cooperation. To visualize the collaborative productivity for each pair of countries, a vector was calculated - proportional in line width and shade of grey to the number of collaborations [19, 22, 24].

### Socioeconomic analysis

In order to assess the contributions of highly active nations in vitamin D research in relation to their socioeconomic status, the countries´ gross domestic products (GDP) were related to their vitamin D research activities as previously described [26]. Economic data were obtained from the *World Economic Outlook Database* of the *International Monetary Fund* [27]. Countries were classified as high-income, upper middle-income, or lower middle-income countries.

### Research areas

By using the original WoS categories, the subject areas of identified articles were analysed in a country specific manner and with regard to their chronological development in 5-year intervals. The author’s key words that have been applied more than 70 times were displayed in a diagram generated by the VOSviewer software [28].

## Results

### General parameters

**Fig. 1:**
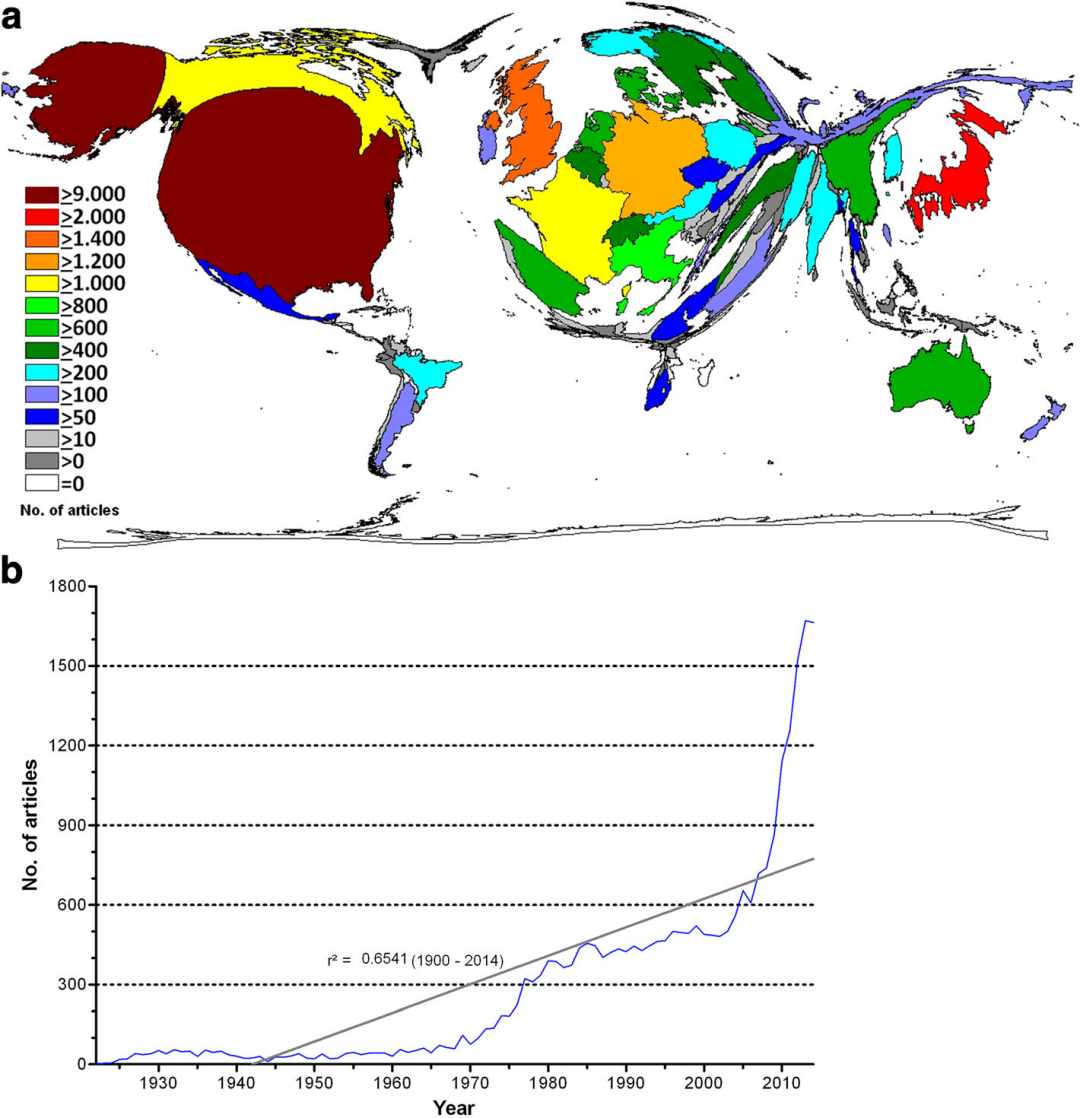
Global vitamin D research activities. **a** Density-equalizing map of numbers of vitamin D articles. The area of each country was scaled in proportion to its total number of publications. **b** Regression analysis of vitamin D articles per year between 1900 and 2014

In total, 25,992 vitamin D-related research articles were found in the selected databases. From 1900 to 2014, regression analysis demonstrates a significant increase (r^2^ = .6541) in published articles. Vitamin D research originates primarily from the USA with a total of 9575 articles (n). We noted a large gap to the next most active country, which is Japan with 2272 vitamin D related publications, followed by the United Kingdom (UK) (*n* = 1855), Germany (*n* = 1240), Canada (*n* = 1183), and France (*n* = 1163). Countries such as Italy (*n* = 800), Australia (*n* = 795), Denmark (*n* = 732), Spain (*n* = 712), the Netherlands (*n* = 636) and China (*n* = 632) had less than 1000 vitamin D related articles. The DEMP-analysis (Fig. 1) illustrates that Northern American authors – particularly authors located in the USA – issued the majority of vitamin D related publications, followed by their Western European counterparts. Africa and South America play only a very minor role in global vitamin D research (Fig. 1).

When we refined the search to synthetic and pharmaceutical forms of vitamin D3, 1.473 Articles (5.6%) were identified. The ranking of the publishing countries remained more or less the same: The USA was identified as the most productive country (*n* = 345), followed by the UK (*n* = 140) and Japan (*n* = 114). The following countries included Germany (*n* = 108), Denmark (*n* = 99), France (n = 99), Italy (*n* = 77), the Netherlands (n = 77), Canada (*n* = 62) and on 10th position Israel (*n* = 51).

### Socioeconomic benchmarking of vitamin D research

**Fig. 2:**
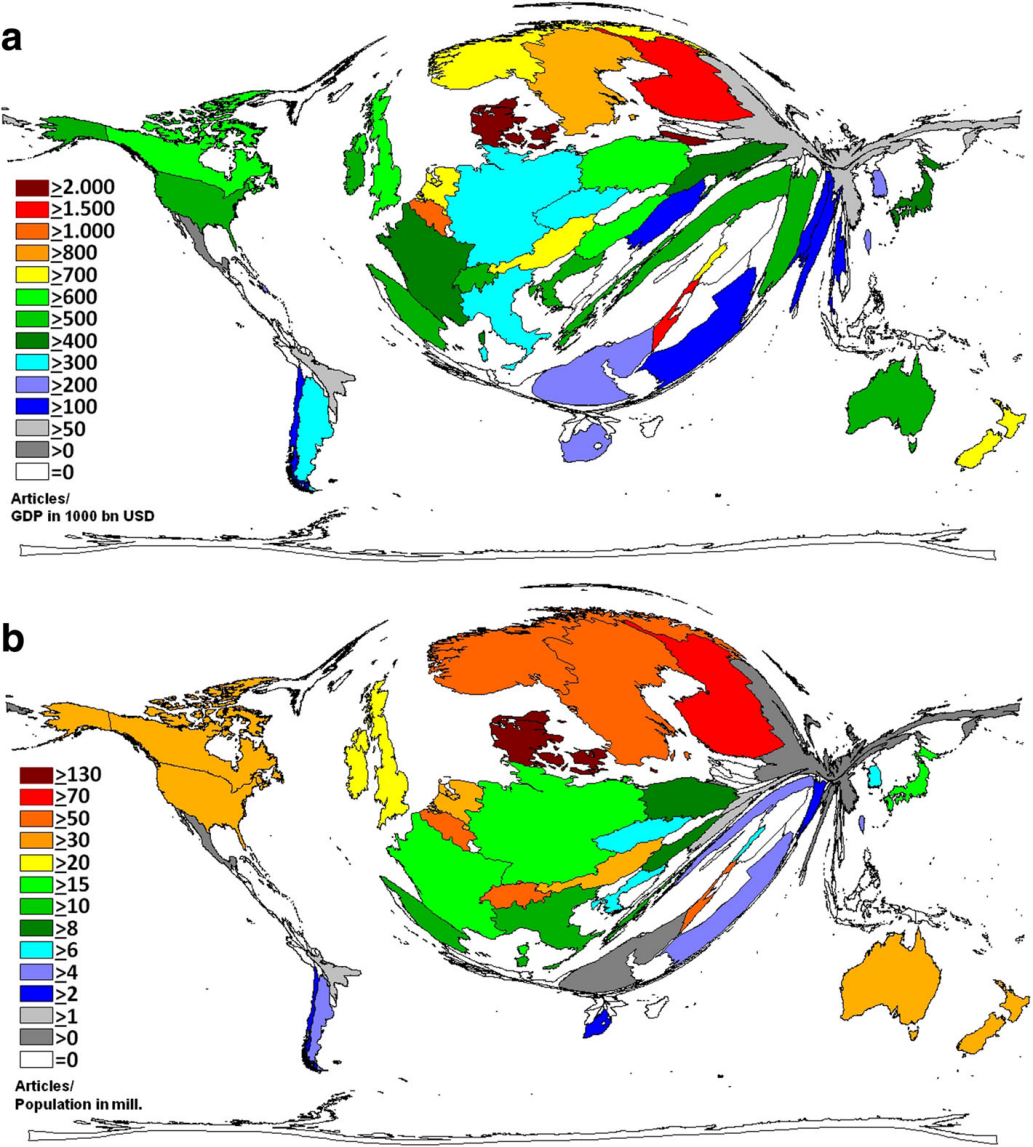
Density-equalizing mapping of socioeconomic features of vitamin D research of global vitamin D research activities (threshold: 30 vitamin D related articles). **a** Vitamin D articles were calculated in relation to GDP in 1000 bn USD. **b** Vitamin D articles were calculated in relation to the population in mill. Inhabitants

Three rankings were conducted by applying socioeconomic characteristics of publishing countries to their global vitamin D research productivity (Table 1): In the 1st ranking, number of vitamin D articles was related to GDP in 1000 billion (bn) USD (R_GDP_). Among high-income countries (HI), Denmark was ranked first with a calculated ratio of 2147.9 R_GDP_. Israel was in second position with a R_GDP_ of 1734.7 followed by Finland (R_GDP_ = 1607.7). By comparison, the USA was characterized only by a R_GDP_ of 549.7, and Japan had a R_GDP_ of 492.2 (Table 1). When this socioeconomic benchmark was applied to upper middle-income (UMI) countries, Lebanon was ranked first with a R_GDP_ of 781.25. However, it needs to be taken into account that the total amount of Lebanese vitamin D articles was just 39 compared to 222 articles by the Iran, which was ranked second UMI country with a R_GDP_ of 549.4. China with a total of 632 articles had a R_GDP_ of only 49.1 (UMI rank # 8). In the group of lower middle-income (LMI) countries, India had a R_GDP_ of 150.7 (LMI rank # 4). It is noteworthy that density equalizing mapping calculations lead to a world map (Fig. 2a) that exhibits large differences in vitamin D research productivity as measured by absolute numbers of articles (see Fig. 1a).

**Table 1:** Socioeconomic features of global vitamin D research. Economic data were obtained from the *World Economic Outlook Database* of the *International Monetary Fund* [27]

	No. of articles	GDP in 1000 bn USD	GDP per Capita	Population total in Mil.	Articles/GDP in 1000 bn USD	Ranking 1	Articles/GDP per Capita	Ranking 2	Articles/Population in Mil.	Ranking 3
USA	9575	17,420	54.8	318.9	549.66	HI18	174.73	HI1	30.03	HI13
Japan	2272	4616	37.8	127.1	492.2	HI21	60.11	HI2	17.88	HI16
Ukraine	1855	2945	37.7	63.74	629.88	HI13	49.2	HI3	29.1	HI15
Germany	1240	3860	44.7	80.99	321.24	HI25	27.74	HI5	15.31	HI18
Canada	1183	1789	44.5	34.83	661.26	HI10	26.58	HI6	33.96	HI11
France	1163	2847	40.4	66.25	408.5	HI22	28.79	HI4	17.55	HI17
Italy	800	2148	34.5	61.68	372.44	HI23	23.19	HI7	12.97	HI20
Australia	795	1444	46.6	22.5	550.55	HI17	17.06	HI9	35.33	HI10
Denmark	732	0.341	44.3	5.56	2147.89	HI1	16.52	HI10	131.65	HI1
Spain	712	1407	33	47.73	506.04	HI20	21.58	HI8	14.92	HI19
Netherlands	636	0.866	47.4	16.87	734.07	HI7	13.42	HI13	37.7	HI9
China	632	10,380	12.9	1355.7	60.89	UMI8	48.99	UMI1	0.47	UMI9
Belgium	540	0.535	41.7	10.44	1009.91	HI4	12.95	HI14	51.72	HI5
Israel	527	0.304	33.4	7.82	1734.69	HI2	15.78	HI11	67.39	HI4
Sweden	488	0.570	44.7	9.72	855.99	HI5	10.92	HI15	50.21	HI7
Finland	436	0.271	40.5	5.26	1607.67	HI3	10.77	HI16	82.89	HI2
Turkey	410	0.806	19.6	81.61	508.62	UMI3	20.92	UMI2	5.02	UMI2
Switzerland	409	0.712	55.2	8.06	574.36	HI14	7.41	HI19	50.74	HI6
Norway	355	0.500	65.9	5.14	709.72	HI8	5.39	HI21	69.07	HI3
Poland	347	0.547	24.4	38.34	634.83	HI12	14.22	HI12	9.05	HI22
South Korea	325	1410	35.4	49.03	230.5	HI28	9.18	HI17	6.63	HI25
Austria	310	0.437	45.4	8.22	709.22	HI9	6.83	HI20	37.71	HI8
India	309	2050	5.8	1236.3	150.73	LMI4	53.28	LMI1	0.25	LMI3
Iran	222	0.404	16.5	80.84	549.37	UMI2	13.45	UMI4	2.75	UMI3
Brazil	206	2353	15.2	202.6	87.55	UMI7	13.55	UMI3	1.02	UMI7
Argentina	194	0.540	22.1	43.02	359.13	HI24	8.78	HI18	4.51	HI30
New Zealand	147	0.198	35	4.4	742.05	HI6	4.2	HI24	33.41	HI12
Saudi Arabia	147	0.753	52.8	27.34	195.35	HI29	2.78	HI28	5.38	HI28
Ireland	141	0.246	46.8	4.83	572.24	HI15	3.01	HI26	29.19	HI14
Greece	133	0.238	25.8	10.77	558.82	HI16	5.16	HI22	12.35	HI21
Russia	124	1857	24.8	142.47	66.77	HI32	5	HI23	0.87	HI32
Taiwan	123	0.530	43.6	23.35	232.25	HI27	2.82	HI27	5.27	HI29
South Africa	96	0.350	12.7	48.37	274.21	UMI4	7.56	UMI5	1.98	UMI4
Hungary	89	0.137	24.3	9.91	649.16	HI11	3.66	HI25	8.98	HI23
Egypt	77	0.286	11.1	86.89	268.85	LMI2	6.94	LMI4	0.89	LMI2
Thailand	73	0.374	14.4	67.74	195.29	UMI5	5.07	UMI6	1.08	UMI6
Czech Republic	64	0.206	28.4	10.62	311.13	HI26	2.25	HI29	6.03	HI26
Ukraine	64	0.131	8.2	44.29	489.67	LMI1	7.8	LMI3	1.45	LMI1
Mexico	63	1283	17.9	120.28	49.1	UMI9	3.52	UMI7	0.52	UMI8
Chile	48	0.258	23.2	17.36	186.05	HI30	2.07	HI30	2.76	HI31
Lebanon	39	0.050	17.9	5.88	781.25	UMI1	2.18	UMI8	6.63	UMI1
Pakistan	39	0.250	4.7	196.2	155.94	LMI3	8.3	LMI2	0.2	LMI4
Romania	35	0.200	19.4	21.72	175	UMI6	1.8	UMI9	1.61	UMI5
Singapore	32	0.308	81.3	5.56	103.86	HI31	0.39	HI32	5.76	HI27
Croatia	31	0.057	20.4	4.47	542.34	HI19	1.52	HI31	6.94	HI24

Countries were classified as high-income, upper middle-income, or lower middle-income countries

As a second socioeconomic benchmark of global vitamin D research activities, we calculated the ratio of the number of vitamin D articles per GDP per Capita in USD (R_GDPpC_) (Table 1): In the HI country ranking, the USA was leading with a R_GDPpC_ of 174.7, followed by Japan (R_GDPpC_ = 60.1), the UK (R_GDPpC_ = 49.2), France (R_GDPpC_ = 28.8), and Germany R_GDPpC_ = 27.7. China was first among the UMI countries (R_GDPpC_ = 50). In the LMI group, we identified India as the leader (R_GDPpC_ of 53.3, Table 1).

The third socioeconomic benchmark of global vitamin D research activity was the quotient of vitamin D-related articles and the population size in mill. Inhabitants (R_POP_) (Table 1): In this ranking, the group of HI countries was led by Denmark with a R_POP_ of 131.7. Number two and three were also Scandinavian countries (Finland and Norway with R_POP_ = 82.9 and R_POP_ = 69.1, respectively). Density equalizing techniques illustrate this dominance of Scandinavian countries (Fig. 2b). The USA was ranked 13th (R_POP_ = 30), the UK 15th (R_POP_ = 29.1), Japan 16th (R_POP_ = 17.9, Table 1). Among UMI countries, Lebanon was ranked first (R_POP_ = 6.6). China had a R_POP_ of 0.47. The Ukraine was the most active LMI country with a R_POP_ of 1.5. The R_POP_ of India was 0.25 (Table 1).

### Vitamin D international collaborative efforts

**Fig. 3:**
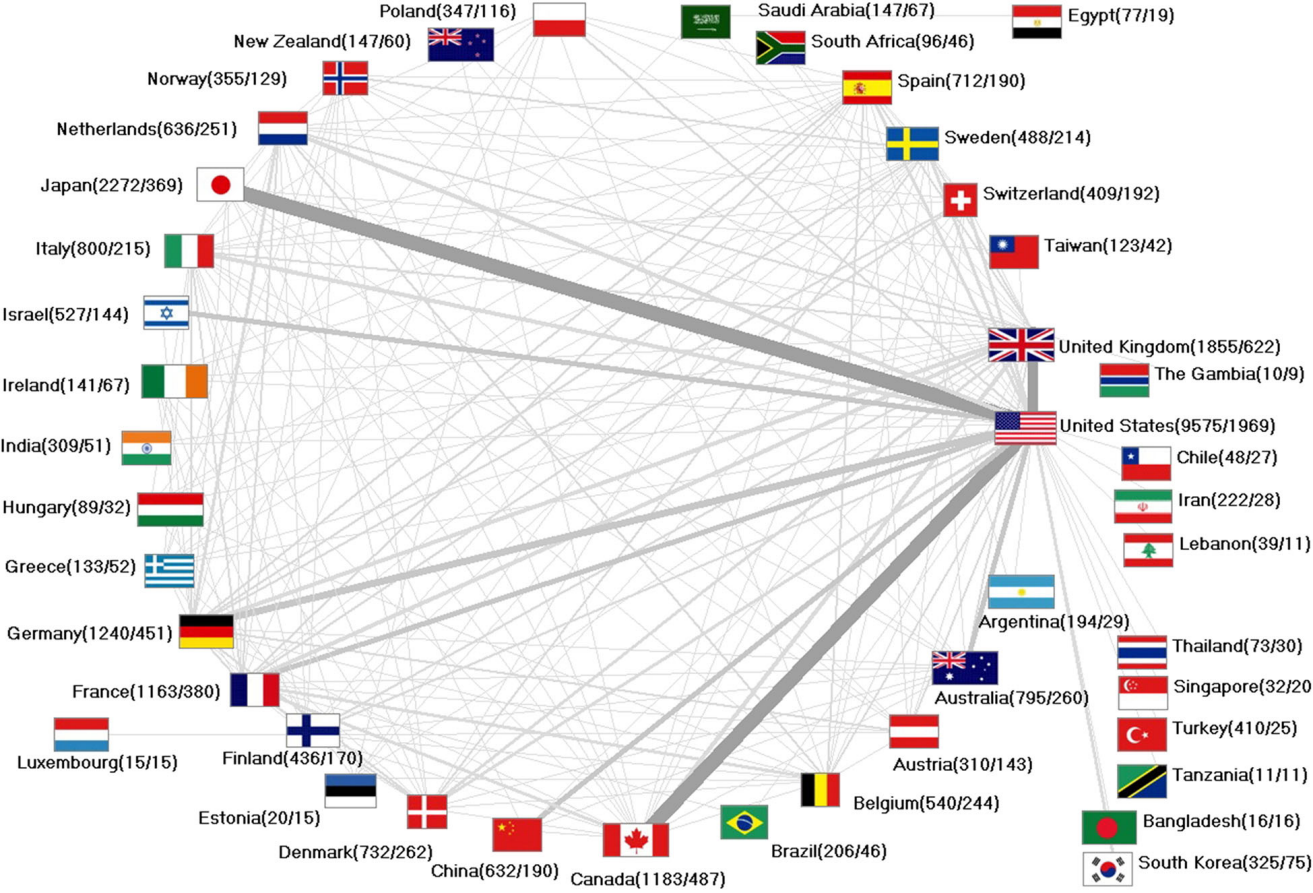
Net diagram of international vitamin D collaborative articles (threshold ≥8 collaborations). Line width and grey scale encode numbers of collaborations. Numbers in brackets (number of publications of a specific country/number of collaboration)

In total, 3467 of all vitamin D articles were a result of an international collaboration between at least two different countries (13.34% of all publications). The most common co-operation type in all collaboration articles was bilateral (*n* = 2821), followed by trilateral (*n* = 436) collaborations. 99 publications were issued by authors from four countries, and for 29 publications, authors from five countries worked together. Twenty-three publications were collaborative efforts by authors from six countries; 14 publications included researchers form seven countries, and 18 articles included scientists from eight countries. Three collaboration articles included authors of 13 countries. A net diagram illustrates the bilateral collaborations (Fig. 3): The USA dominates all bilateral collaborative efforts with 1969 collaboration articles among its 9575 vitamin D publications. The majority of these collaborations were established between USA and Canada, USA and the UK and USA and Japan. Among the most active countries in vitamin D research, Canada was leading the overall collaborative efforts with 487 of its 1183 articles being issued within a network of international co-operations.

### Vitamin D research areas

**Fig. 4:**
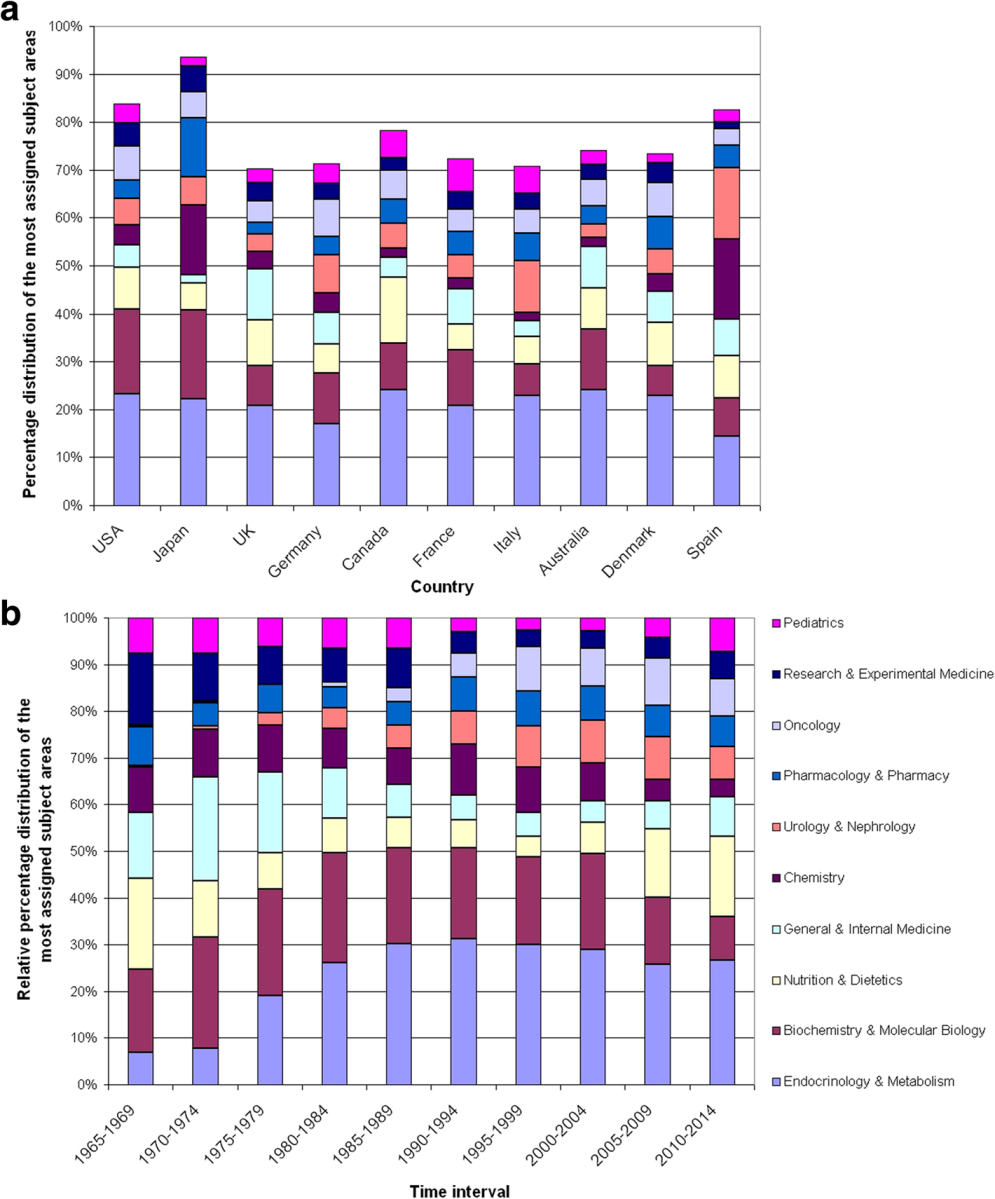
Subject areas activity. **a** Proportion of the 10 most assigned subject areas in five-year intervals in order to gain insights into the field activity. **b** Country-specific proportion of the 10 most assigned subject areas in countries with the highest osteoporosis research activity

Vitamin D research areas were analysed using standard subject categories assigned to the articles by the WoS. Evolution over time was monitored using the subject area as a proportion of the total articles published in five-year intervals (Fig. 4a). This analysis showed an increase of the subject area ‘*Endocrinology & Metabolism*’ from 1965 (less than 10%) to 1994 (over 30%). The category ‘*Biochemistry and Molecular Biology*’ is characterized by a decrease in research activity over the past decades with a proportion of more than 20% in the 1980s to less than 10% from 2010 onwards. Interestingly, the area ‘*Nutrition and Dietetics*’ had a high proportion in 1965–1969, decreased afterwards and gained a relatively high proportion after 2005.

In order to identify country specific focuses of vitamin D research, we analysed the proportion of subject areas in the ten leading countries and found major differences (Fig. 4b): While ‘*Endocrinology & Metabolism*’ was the most popular subject area in all countries, Canada was characterized by a special research interest in ‘*Nutrition and Dietetics*’ with about 14% of all its articles focused on this field. Less than 2% of Canadian articles were related to ‘*Chemistry*’. By contrast, 14.6% of Japanese and 16.7% of Spanish vitamin D research were carried out in *‘Chemistry’*. Among clinical areas, the field of ‘*Urology and Nephrology*’ was prominent in Italian and Spanish vitamin D articles with 10.8% and 14.9%, respectively (Fig. 4b).

**Fig. 5:**
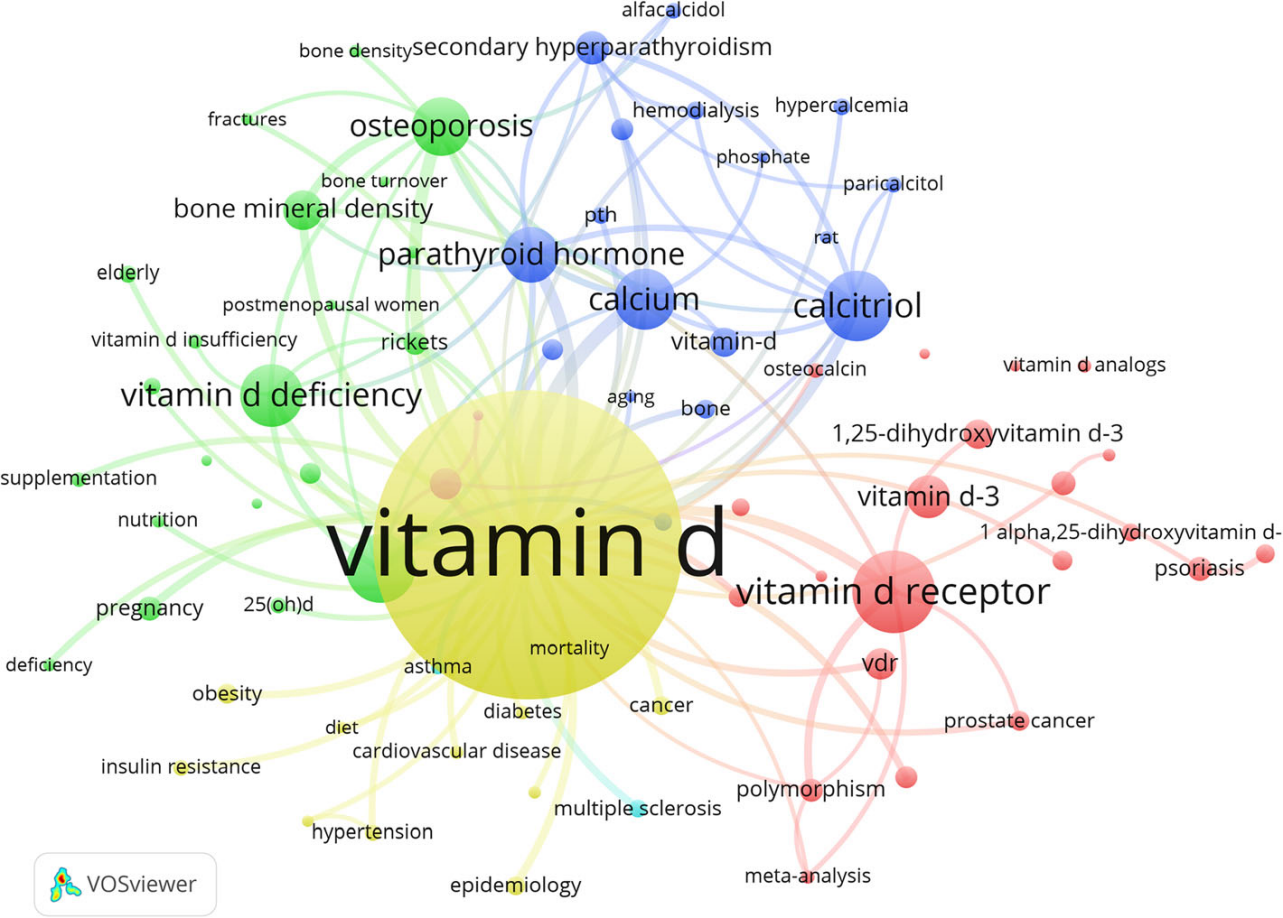
Main key word analysis. Analysis of key words, which were attributed for a minimum of 70 times

We analysed the research fields according to the authors’ keywords. Here, four main thematic clusters exist (Fig. 5). On the one hand, authors focused on the health consequences caused by vitamin D deficiency and risk factors for deficiency, respectively the risk factors. On the other hand, the different chemical forms of vitamin D (e.g. the synthetic type) and their implications for health were also popular among scientists.

### Country citation analysis

**Fig. 6:**
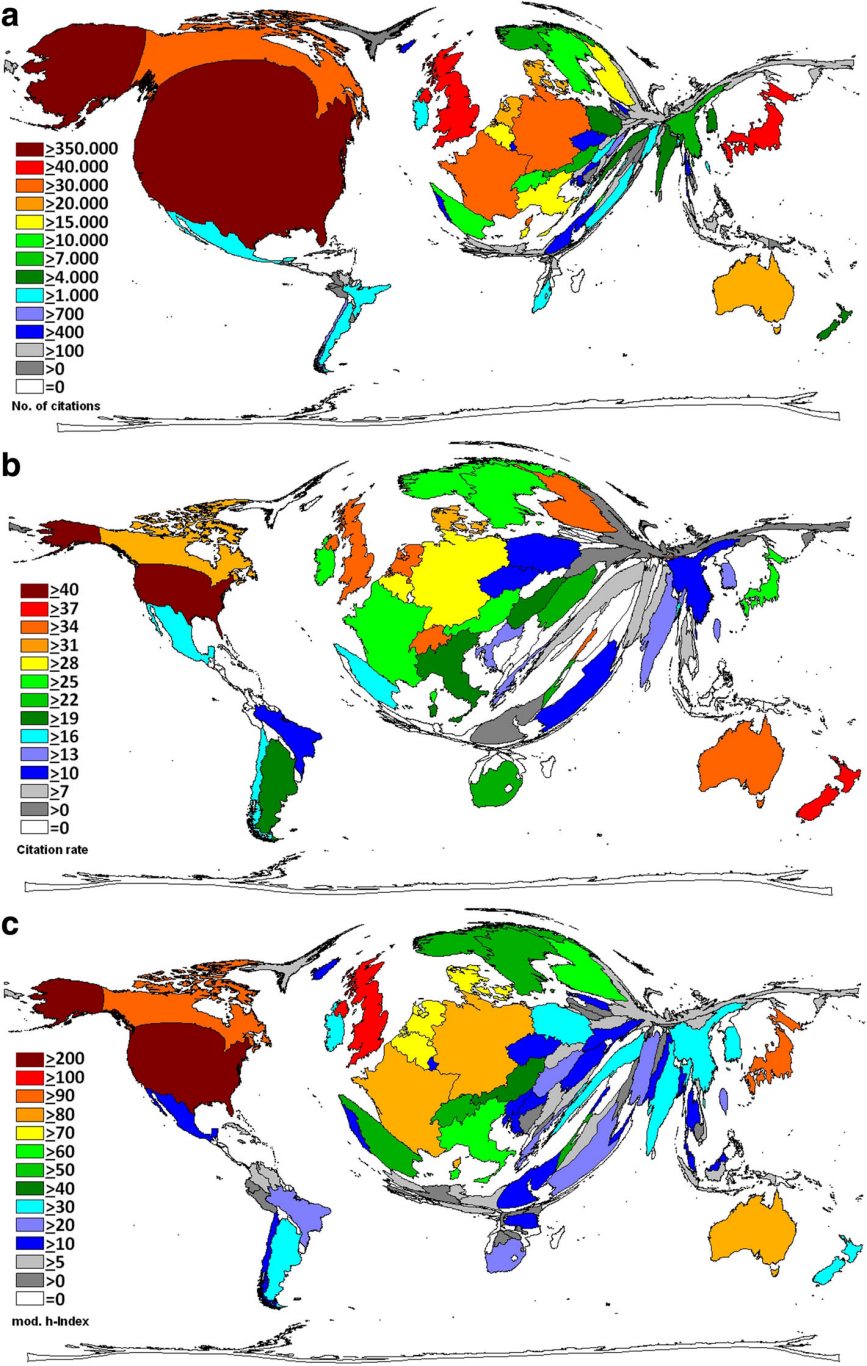
Citation analysis. **a** Density-equalizing map of country total citations. The area of each country was scaled in proportion to its total number of citations. **b** Density-equalizing map of country citation rates (threshold: 30 vitamin D related articles). The area of each country was scaled in proportion to citations per published article rate. **c** Density-equalizing map of country-specific h-indices (modified). The area of each country was scaled in proportion to country-specific h-indices

In the overall citation analysis (Fig. 6a), the USA again was the leading country with 394,625 citations (c) attributed to its vitamin D articles. The USA was followed by the UK with 68,463 citations. Japanese vitamin D articles were cited 58,054 times, followed by articles from Canada (c = 39,024), Germany (c = 36,159) and France (c = 31,757). The country citations rate (citations per publication of a country, cr) is a semi-qualitative measure and was applied to countries with at least 30 vitamin D related articles. Here, the USA was leading the field with a cr of 41.21, followed by New Zealand (cr = 38.7), and the UK (cr = 36.9). Articles from the Netherlands had 36.4 citations per vitamin D article; Australia had a cr of 35.2, followed by Germany (cr = 29.2), Japan (cr = 25.6), and China (cr = 11.3, Fig. 6b). When the country-specific h-index was calculated, the USA had a h-index (hi) of 223 (i.e.223 articles were being cited at least 223 times). The USA was followed by the UK (hi = 115), Japan (hi = 95), Canada (hi = 93), Germany (hi = 87), France (hi = 82) and Australia (hi = 80). China had a h-index of 37 (Fig. 6c).

## Discussion

Although vitamin D is an important micronutrient that has been associated with various diseases including neoplasms such as breast cancer or prostate cancer [29–33], yet no assessment of the global research productivity related to vitamin D has been conducted so far. Since vitamin D does not only play a major role not only in nutrition but also in areas such as musculoskeletal medicine, gynaecology or oncology, the concise analysis of global research efforts targets multiple fields and is of interest to a broad spectrum of scientists. Therefore, the present initiative was started by experts in vitamin D research and scientometrics to conduct an in-depth analysis of the existing global research landscape. Using the established NewQIS study protocol, the present study combines novel visualization techniques such as density-equalizing mapping [25] and scientometrics [12].

Prior to the discussion of the results, several methodological issues need to be addressed and taken into account when interpreting the data. As primary database, we chose the Web of Science (WoS) over PubMed since the WoS enabled us to assess citations in a standardized way. We acknowledge that different numbers of vitamin D related articles would be identified when searching for identical terms in WoS and PubMed, since the two databases include different sets of scientific journals. This points also to the fact that every literature search has its limitations and will not cover all items ever published on a specific topic of interest. In this respect, the language bias needs to be discussed. As with all English literature databases, the WoS has a clear preference for journals published in English. Hence, numerous vitamin D related articles published in non-English scientific journals were not identified by our search. Perhaps, the recent addition of the Emerging Sources Citation Index to the WoS is a response of the database provider to address this problem. The Emerging Sources Citation Index integrates additional high-quality, peer-reviewed publications of regional importance into the WoS. This emphasis on relevant regional journals will result in a higher quantity of non-English publications indexed in this database. Regardless of the number of regional, non-English language journals, it can be assumed that high quality articles are typically published in international journals. These journals tend to have high impact factors and are usually included in the WoS. Therefore, the present study should encompass nearly all highly relevant articles related to vitamin D.

In our study, we identified a total number of 25,992 vitamin D articles which have been authored from 1900 to 2014. In order to correctly assess the results, we provide a comparison to another intriguing and common medical issue, gestational diabetes, that has been evaluated similarly. In the period from 1900 to 2012, only 12,504 articles on gestational diabetes were identified in the same database [12]. While the global output on vitamin D was approximately double that on gestational diabetes, the relative global distribution was about the same, with a domination by industrialized North-American, and Western European nations [12]. Concerning Japan, there were differences in both research areas. While Japan was the second most active country in vitamin D research with 2272 publications between 1900 and 2014, it had less than 300 articles related to gestational diabetes [12]. This finding may due to the low prevalence of GDM in Japan, which is documented at 2.9% [34].

Apart from recent years in which the association between vitamin D and its receptors and different neoplasms were widely discussed [35–39], the micronutrient vitamin D has been studied as a key player in the prevention of osteoporosis [40, 41]. Therefore, it is enticing to speculate whether the global vitamin D research landscape largely parallels the osteoporosis research landscape. A recently published study, that used the identical platform, identified 57,453 entries related to osteoporosis authored from 1900 to 2012 [22–26]. The USA was the most active county with 18,512 osteoporosis publications, followed by the UK, Germany, Japan, France and Canada. Hence, a similar distribution of productivity was found for osteoporosis and vitamin D research; the top six productive countries were the same, but appeared in a different order. Although an association between the two scientific fields could be assumed, we hypothesize that the similarity most likely depend on the general publishing power of these respective countries. We want to underline this statement by a previous benchmarking study, which analyzed 5,527,558 publications on 21 organ systems that were authored from 1961 to 2007. Here, the USA was the most productive nation and issued 1,893,800 publications. UK, Germany, Japan, France and Canada – the major key players in vitamin D and osteoporosis research – were also found among the seven most productive nations in 21 biomedical fields [42].

The relative resource allocation of single countries to vitamin D research is of major interest for the scientific community and health services system research. For this, we employed three socioeconomic benchmarks to the field of vitamin D research, which consisted of the following ratios: Research output 1) to the countries´ GDP in 1000 bn USD, 2) to country GDP per Capita, and 3) to the population size. The world map of the scientific productivity in relation to socioeconomic aspects changed dramatically in comparison to the maps depicting absolute quantity or quality of evaluated research. Clearly, Scandinavian countries – with Denmark in the leading position – dominated two of the three socioeconomic benchmarks. This finding reflects the strong dedication of these countries to vitamin D research. Science is done with high efficiency since Scandinavian researchers generate the highest quantity of articles in relation to their nations’ economic power and scientific manpower. The dominance of Scandinavian countries in this particular analysis is largely similar to their dominance regarding other medical topics such as ovarian cancer [43] or maternal depression research, which are areas with great importance for public health [43].

Overall, vitamin D hypovitaminosis is a global problem, which is prevalent in almost every region of the world. That is why significance must be attributed to this matter in relation to many health issues worldwide. Many important research topics remain and need to be addressed such as the paucity of epidemiological data from Africa or parts of Asia [6], reliable and accurate assay methods, and differences in vitamin D metabolism by ethnicity. Research needs to be fostered by funding and collaborative networks, which should include countries with a low vitamin D status to exchange data and gain knowledge together. The vitamin D standardization program (VDSP) is an excellent example of a state of the art approach to the problems encountered [44–46].

## Conclusions

We here present the first analysis of the global research productivity on vitamin D. We assessed all articles related on this topic that have been published since 1900 in the WoS.

Scientific networks dedicated to Vitamin D research have been established around the globe. Here, high-income countries such as the USA are authoring the majority of high impact articles and spearheading the leading number of collaborations. However, we identified gaps within the global scientific community: South American, African and Asian countries apart from Japan only play a very minor role in worldwide activities. Since awareness of vitamin D status is currently increasing in the Americas, Europe and parts of the Middle East [47], funding agencies should focus on vitamin D research in these regions.
