# Correction to: World-wide research architecture of vitamin D research: density-equalizing mapping studies and socio-economic analysis

**DOI:** 10.1186/s12937-018-0339-9

**Published:** 2018-02-20

**Authors:** Dörthe Brüggmann, Annahita Allafi, Jenny Jaque, Doris Klingelhöfer, Michael H. Bendels, Daniela Ohlendorf, David Quarcoo, Frank Louwen, Sue A. Ingles, Eileen M. Wanke, David A. Groneberg

**Affiliations:** 1 Department of Gynecology and Obstetrics, Goethe-University, Frankfurt, Germany; 2 Division of Preventive Medicine, Institute of Occupational Medicine, Social Medicine and Environmental Medicine, Goethe-University, Frankfurt, Germany; 3 Department of Obstetrics and Gynecology, Keck School of Medicine, University of Southern California, Los Angeles, CA USA; 4 Department of Preventive Medicine, University of Southern California, Los Angeles, USA

## Correction

After publication of the original article [1], the authors reported two errors which needed to be corrected:

The Family Name of Annahita Allafi was misspelled as Alafi. The correct presentation of the author’s name is displayed in the author list of this Correction article.

In Table 1, “Ukraine” should be “United Kingdom” in the third row of the column entitled “Country”. The correct version of Table 1 is included in this Correction article.

**Table 1:** ᅟ

Country	No. of articles	GDP in 1000 bn USD	GDP per Capita	Population total in Mil.	Articles/GDP in 1000 bn USD	Ranking 1	Articles/GDP per Capita	Ranking 2	Articles/Population in Mil.	Ranking 3
United States	9575	17,420	54,80	318,90	549,66	HI18	174,73	HI1	30,03	HI13
Japan	2272	4616	37,80	127,10	492,20	HI21	60,11	HI2	17,88	HI16
United Kingdom	1855	2945	37,70	63,74	629,88	HI13	49,20	HI3	29,10	HI15
Germany	1240	3860	44,70	80,99	321,24	HI25	27,74	HI5	15,31	HI18
Canada	1183	1789	44,50	34,83	661,26	HI10	26,58	HI6	33,96	HI11
France	1163	2847	40,40	66,25	408,50	HI22	28,79	HI4	17,55	HI17
Italy	800	2148	34,50	61,68	372,44	HI23	23,19	HI7	12,97	HI20
Australia	795	1444	46,60	22,50	550,55	HI17	17,06	HI9	35,33	HI10
Denmark	732	0,341	44,30	5,56	2147,89	HI1	16,52	HI10	131,65	HI1
Spain	712	1407	33,00	47,73	506,04	HI20	21,58	HI8	14,92	HI19
Netherlands	636	0,866	47,40	16,87	734,07	HI7	13,42	HI13	37,70	HI9
China*	632	10,380	12,90	1355,70	60,89	UMI8	48,99	UMI1	0,47	UMI9
Belgium	540	0,535	41,70	10,44	1009,91	HI4	12,95	HI14	51,72	HI5
Israel	527	0,304	33,40	7,82	1734,69	HI2	15,78	HI11	67,39	HI4
Sweden	488	0,570	44,70	9,72	855,99	HI5	10,92	HI15	50,21	HI7
Finland	436	0,271	40,50	5,26	1607,67	HI3	10,77	HI16	82,89	HI2
Turkey	410	0,806	19,60	81,61	508,62	UMI3	20,92	UMI2	5,02	UMI2
Switzerland	409	0,712	55,20	8,06	574,36	HI14	7,41	HI19	50,74	HI6
Norway	355	0,500	65,90	5,14	709,72	HI8	5,39	HI21	69,07	HI3
Poland	347	0,547	24,40	38,34	634,83	HI12	14,22	HI12	9,05	HI22
South Korea	325	1410	35,40	49,03	230,50	HI28	9,18	HI17	6,63	HI25
Austria	310	0,437	45,40	8,22	709,22	HI9	6,83	HI20	37,71	HI8
India	309	2050	5,80	1236,30	150,73	LMI4	53,28	LMI1	0,25	LMI3
Iran	222	0,404	16,50	80,84	549,37	UMI2	13,45	UMI4	2,75	UMI3
Brazil	206	2353	15,20	202,60	87,55	UMI7	13,55	UMI3	1,02	UMI7
Argentina	194	0,540	22,10	43,02	359,13	HI24	8,78	HI18	4,51	HI30
New Zealand	147	0,198	35,00	4,40	742,05	HI6	4,20	HI24	33,41	HI12
Saudi Arabia	147	0,753	52,80	27,34	195,35	HI29	2,78	HI28	5,38	HI28
Ireland	141	0,246	46,80	4,83	572,24	HI15	3,01	HI26	29,19	HI14
Greece	133	0,238	25,80	10,77	558,82	HI16	5,16	HI22	12,35	HI21
Russia	124	1857	24,80	142,47	66,77	HI32	5,00	HI23	0,87	HI32
Taiwan	123	0,530	43,60	23,35	232,25	HI27	2,82	HI27	5,27	HI29
South Africa	96	0,350	12,70	48,37	274,21	UMI4	7,56	UMI5	1,98	UMI4
Hungary	89	0,137	24,30	9,91	649,16	HI11	3,66	HI25	8,98	HI23
Egypt	77	0,286	11,10	86,89	268,85	LMI2	6,94	LMI4	0,89	LMI2
Thailand	73	0,374	14,40	67,74	195,29	UMI5	5,07	UMI6	1,08	UMI6
Czech Republic	64	0,206	28,40	10,62	311,13	HI26	2,25	HI29	6,03	HI26
Ukraine	64	0,131	8,20	44,29	489,67	LMI1	7,80	LMI3	1,45	LMI1
Mexico	63	1283	17,90	120,28	49,10	UMI9	3,52	UMI7	0,52	UMI8
Chile	48	0,258	23,20	17,36	186,05	HI30	2,07	HI30	2,76	HI31
Lebanon	39	0,050	17,90	5,88	781,25	UMI1	2,18	UMI8	6,63	UMI1
Pakistan	39	0,250	4,70	196,20	155,94	LMI3	8,30	LMI2	0,20	LMI4
Romania	35	0,200	19,40	21,72	175,00	UMI6	1,80	UMI9	1,61	UMI5
Singapore	32	0,308	81,30	5,56	103,86	HI31	0,39	HI32	5,76	HI27
Croatia	31	0,057	20,40	4,47	542,34	HI19	1,52	HI31	6,94	HI24
